# Thermodynamic insights into selenium oxyanion removal from synthetic flue gas desulfurization wastewater with temperature-swing solvent extraction

**DOI:** 10.3389/fchem.2023.1225843

**Published:** 2023-09-08

**Authors:** Michael S. Meissner, Vy H. T. Nguyen, Imen Bousrih, Van T. C. Le, Alex Frickenstein, Giang V. Le, Ngoc T. Bui

**Affiliations:** ^1^ School of Chemical, Biological, and Materials Engineering, The University of Oklahoma, Norman, OK, United States; ^2^ Stephenson School of Biomedical Engineering, Norman, OK, United States; ^3^ Central Institute for Natural Resources and Environmental Studies, Vietnam National University, Hanoi, Viet Nam; ^4^ School of Civil Engineering and Environmental Science, The University of Oklahoma, Norman, OK, United States

**Keywords:** selenate, selenite, mercury, deionization, zero liquid discharge

## Abstract

Temperature-swing solvent extraction (TSSE) is a cost-effective, simple, versatile, and industry-ready technology platform capable of desalinating hypersaline brines toward zero liquid discharge. In this work, we demonstrate the potential of TSSE in the effective removal of selenium oxyanions and traces of mercury with the coexistence of high contents of chloride and sulfate often encountered in flue gas desulfurization wastewater streams. We compare the rejection performance of the two common solvents broadly used for TSSE, decanoic acid (DA) and diisopropylamine (DPA), and correlate those with the solvent physicochemical properties (e.g., dielectric constant, polarity, molecular bulkiness, and hydrophobicity) and ionic properties (e.g., hydrated radii and H-bonding). The results show that TSSE can remove >99.5% of selenium oxyanions and 96%–99.6% of mercury traces coexisting with sulfate (at a sixfold Se concentration) and chloride (at a 400-fold Se concentration) in a synthetic wastewater stream. Compared to diisopropylamine, decanoic acid is more effective in rejecting ions for all cases, ranging from a simple binary system to more complex multicomponent systems with highly varied ionic concentrations. Furthermore, the H-bonding interaction with water and the hydrated radii of the oxyanions (i.e., selenate vs*.* selenite) along with the hindrance effects caused by the molecular bulkiness and hydrophobicity (or lipophilicity) of the solvents play important roles in the favorable rejection of TSSE. This study shows that TSSE might provide a technological solution with a high deionization potential for the industry in complying with the Environmental Protection Agency regulations for discharge streams from coal-fired power facilities.

## 1 Introduction

Despite being a major contributor to global electricity production, coal-fired power plants (CFPPs) contribute to climate change and induce adverse environmental impact due to air emissions (e.g., carbon dioxide, nitrogen oxides, and sulfur dioxide) (Hu et al., 2000; Bürkle et al., 2018; He et al., 2021a; He et al., 2021b) and aqueous emissions of heavy metals and other bioaccumulative pollutants at these facilities. In the United States, coal accounted for 21.8% of electricity production in 2021, and approximately 25% of the currently operating U.S. coal-fired capacity is due to retire by the end of 2029 (EIA, 2022). While phasing out, effective pollution control is essential for CFPPs to comply with the Effluent Limitations Guidelines (ELGs) for the Steam Electric Power Generating Sector standards. For instance, to limit SO_2_ emissions, a toxic environmental pollutant that primarily effectuates acid rain (Kaminski, 2003; Wang and Anthony, 2008), CFPPs are obliged to implement flue gas desulfurization (FGD) techniques to gaseous waste streams before atmospheric release (Srivastava et al., 2001; Kaminski, 2003). These techniques with the division into once-through and regenerative approaches are generally employed under wet (e.g., scrubbing with Ca-, Mg-, and NH_3_-based compounds), semi-dry (e.g., using an Na- or Zn-based sorbent), or dry (sorbent injection, circulating fluidized bed, zeolite adsorption, etc.) conditions (Srivastava et al., 2001; Chen et al., 2019; Hanif et al., 2020). Of which, the most commercially profitable practice for FGD in industrial applications is to bring flue gas in contact with a limestone slurry in a wet scrubbing system to capture SO_2_ into the aqueous phase as gypsum (CaSO_4_·2H_2_O) (Carletti et al., 2015; Chen et al., 2019).

Furthermore, it is reported that coal deposits in the earth are contaminated with several trace elements, such as selenium, mercury, and arsenic (Cheng et al., 2009), present at the site of coal formation or delivered via groundwater cycles (Ketris and Yudovich, 2009; Vejahati et al., 2010). Under combustion, these trace elements are released from the coal feeds (Skalnaya and Skalny, 2018; Mehri, 2020) and are, subsequently, partitioned into FGD wastewaters. FGD wastewaters are commonly disposed of via environmental release after treatment (Agency, 2015), turning coal combustion into one of the principal culprits of environmental selenium pollution (Ohlendorf et al., 2011; Gingerich et al., 2018; He et al., 2018). The Environmental Protection Agency (EPA) updated the effluent limits for environmental discharge of FGD waters in 2015 (EPA, 2023), yet engineering challenges involved in meeting the new discharge standards caused the EPA to postpone the compliance date for existing CFPPs (EPA, 2017). In March 2023, the EPA proposed the establishment of more stringent discharge standards for CFPP wastewater (including Se, Hg, As, Ni, and halogen compounds), which would potentially reduce the amount of pollutants discharged through these streams by approximately 584 million pounds per year (Coleman, 2023; EPA, 2023).

It is noteworthy that approximately 30% of selenium found in coal feeds partitions into FGD wastewater (Cheng et al., 2009). These high levels of selenium are reported to pose threats to the environment (Hamilton, 2004; Gingerich et al., 2018) and human health (Skalnaya and Skalny, 2018; Mehri, 2020). The two main species of selenium that exist in the aquatic environment are water-soluble selenate (SeO_4_^2−^) and selenite (SeO_3_^2−^) (He et al., 2018; Meher et al., 2020). Removing these selenium oxyanions from water remains challenging (Gingerich et al., 2018), especially the removal of the former ion due to its kinetically non-reactive behavior along with its structural similarity to sulfate (SO_4_^2−^)—a co-contaminated anion in most circumstances (Ali and Shrivastava, 2021). Often, sulfate coexists in FGD wastewater at an order of magnitude more prevalent than selenate, which dominates the treatment process, and thus reduces the selenate removal efficiency (Huang et al., 2013; Tokunaga and Takahashi, 2017). Note that FGD wastewater also contains high chloride contents (ranging from 20,000 to 40,000 mg/L), necessitating treatment systems that can effectively function in corrosive environments having high amounts of total dissolved solids (EPA, 2009; Gingerich et al., 2018).

Current industrial efforts for selenium removal are mainly based on biological and chemical approaches, whereas physical methods (e.g., nanofiltration and reverse osmosis) have not captured much attention from industries due to the operational and maintenance costs (Ali and Shrivastava, 2021). Some advanced bioreactors, such as inverse fluidized bed bioreactors, granular sludge reactors, and hybrid bioreactors, displayed excellent Se-reducing bacterial activities, followed by the moderate recovery efficiency of selenium nanoparticles in a single-stage system (Cordoba and Staicu, 2018; Sinharoy and Lens, 2020; Sinharoy et al., 2022). Nevertheless, high sensitivity to variations in feed components and long operating periods of bioremediation unfavorably hinder its practical applications (Gingerich et al., 2018). Coagulation/precipitation, ion exchange, separation, and adsorption dominate the conventional chemical technologies for oxyanion removal. For instance, nano-Al_2_O_3_ embedded in chitosan beads were reported to be able to combine photooxidation with adsorption to synergistically remove selenate and selenite; however, their performances were moderately thwarted with the presence of competing sulfate ions (Pincus et al., 2019). Co-precipitation of selenium oxyanions with barite followed by a phosphate post-treatment step to minimize selenium leakage in different aqueous environments was also reported (Tokunaga et al., 2023). Other common practices for the concurrent removal of selenate and selenite involve the use of nanosized zero-valent iron (nZVI). In these processes, supplementary oxidants are not required, as selenite inherently activated the reactivity of nZVI, essentially enhancing the removal rate and electron selectivity of selenate (Wu et al., 2021). Recently, electrochemical processes have also been leveraged to drive the conversion of these anions to more treatable forms of selenium through different redox pathways (MeaganMauter, 2021; Zou et al., 2021; Zou and Mauter, 2021). Still, the low tolerance of these technologies with the high range of sulfate and chloride often encountered in FGD wastewater streams may have hindered their widespread use, especially where a pre-treatment process is not equipped. Essentially, while the coexisting sulfate may compete with selenium oxyanions for electrons in cathodic parasitic reactions (Zou et al., 2021), chloride may disrupt Se(IV) reduction pathways by generating strong oxidants on the anode side of the process.

**FIGURE 1:**
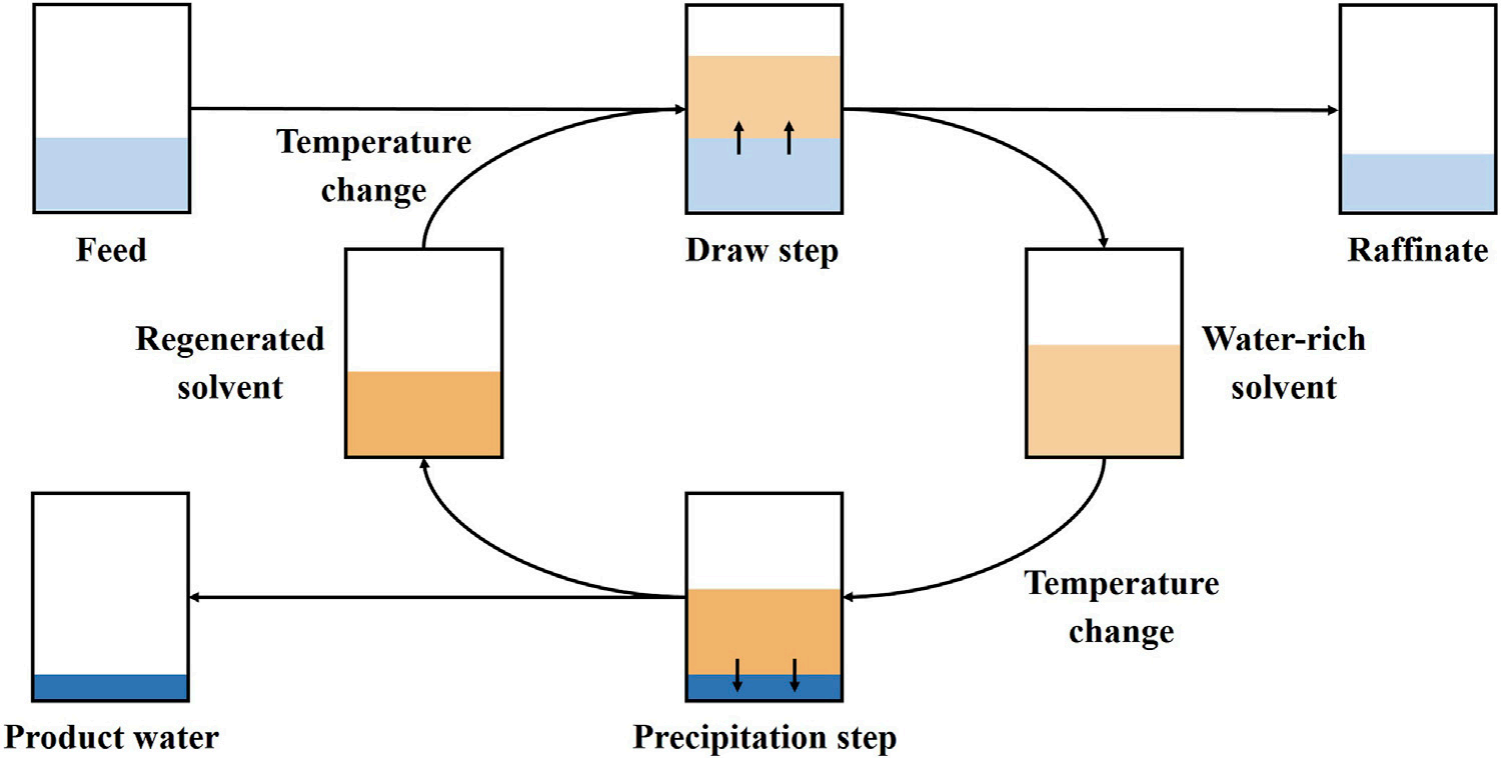
General TSSE process diagram.

Solvent extraction is a versatile and effective non-evaporative separation process that has been applied in various applications due to its relatively low cost and simplicity, including CO_2_ capture, bio-oil fractionation, extraction of metal complexes, and desalination, to name a few (Kumar et al., 2015; Zhang et al., 2022). Temperature-swing solvent extraction (TSSE) has been recently developed, mainly to meet the incremental demand in desalinating water, with its working principle based on the high thermal sensitivity of water solubility of certain solvents (Zhang et al., 2022). Figure 1 provides a diagram of a standard TSSE process. Essentially, an aqueous feed is equilibrated with an organic solvent at a specific temperature favoring water dissolution into the organic solvent while rejecting other compounds. This creates two distinguished phases, *viz*., a concentrated raffinate and a water-rich solvent. The latter is then brought to equilibrium at a different temperature that promotes the immiscibility between the water and organic solvent. Finally, phases split, from which the water product is recovered in the aqueous phase, while the organic solvent can be recycled in subsequent extractions and sustainably reused in a recyclable solvent loop, which averts the usage of a large volume of organic extractants, mitigating their influences on the environment (Bajpayee et al., 2011).

Certain gaps in current studies on the employment of the TSSE method and its targeted objectives have been recognized. The TSSE literature has primarily focused on the organic solvents of diisopropylamine (DPA) (Boo et al., 2019; Boo et al., 2020; Sappidi et al., 2021; Zhang et al., 2022) and decanoic acid (DA) (Bajpayee et al., 2011; Rish et al., 2014; Guo et al., 2020). TSSE with DA was shown to attain high rejections (>98%) of all major ions in seawater including Na^+^, K^+^, Ca^2+^, Mg^2+^, Cl^−^, and SO_4_^2-^, even in the presence of multiple monovalent and divalent cations (Rish et al., 2014), while a single pass of TSSE exhibited the removal efficiencies of 91% for As(III) and 97% for As(V) (Guo et al., 2020). Meanwhile, DPA was reported with the capability of treating feeds containing 4.0 M NaCl (Boo et al., 2019) and impressively achieving zero liquid discharge (Boo et al., 2020). Notwithstanding, apart from a dearth of insight about physicochemical interaction mechanisms between solute–solute and solute–solvent in the TSSE process, how these solvent systems behave in multicomponent feeds with diverse ion concentrations is still unclear. The impact of factors such as solvent physicochemical profiles and ion properties (e.g., hydration radius and atomic charge) on TSSE separating performance has also not been clarified (Rish et al., 2014). On top of that, the TSSE rejection potential of selenate and selenite, particularly from FGD wastewater produced in CFPPs, has not been thoroughly explored to date.

In this work, we demonstrate the potential of TSSE in the effective removal of selenium oxyanions and mercury traces with the coexistence of high contents of chloride and sulfate often encountered in flue gas desulfurization wastewater streams. We compare the rejection performances of the two common solvents broadly used for TSSE, decanoic acid (DA) and diisopropylamine (DPA), and correlate those with the solvent physicochemical properties (e.g., dielectric constant, polarity, molecular bulkiness, and hydrophobicity) and ionic properties (e.g., hydrated radii and H-bonding). We challenge TSSE to remove selenium oxyanions and mercury traces from complex water streams having a high concentration of competing ions, for example, sulfate (at a sixfold Se concentration) and chloride (at a 400-fold Se concentration). Despite being extensively utilized in liquid–liquid extraction, diisopropylamine and decanoic acid behaviors and their chemical interactions with solutes have rarely been inspected thermodynamically at the molecular level, especially in complex multicomponent systems with highly varied ionic concentrations. Furthermore, we present the important roles of H-bonding interactions with water and the hydrated radii of the oxyanions (i.e., selenate vs*.* selenite) along with the hindrance effects caused by the solvent bulkiness and hydrophobicity (or lipophilicity) in the rejection tendency of TSSE. The results provide profound thermodynamic insights into the removal of selenium oxyanions from complex water streams using the TSSE technology platform.

## 2 Experimental

### 2.1 Chemicals

Diisopropylamine (C_6_H_15_N, ≥99.5%), sodium selenate (Na_2_SeO_4_, BioXtra), sodium selenite (Na_2_SeO_3_, 99%), mercury(II) chloride (HgCl_2_, ≥99.5%), decanoic acid (C_10_H_20_O_2_, ≥99.5%), and calcium sulfate dihydrate (CaSO_4_·2H_2_O, ≥99%) were purchased from MilliporeSigma. Sodium chloride (NaCl, certified ACS, crystalline) was purchased from Fisher Chemical. For inductively coupled plasma-optical emission spectrometry (ICP-OES) and/or inductively coupled plasma mass spectrometry (ICP-MS) analyses, selenium standard (1 mg L^−1^ Se in nitric acid), mercury standard (1,000 mg L^−1^ Hg in nitric acid), and gold standard (1,000 mg L^−1^ in hydrochloric acid) were purchased from MilliporeSigma; 28-element ICP calibration/quality control standard and scandium standard (1,000 μg mL^−1^ in 7% nitric acid) were purchased from Inorganic Ventures; and yttrium standard (1,000 μg mL^−1^ in 2% nitric acid) was purchased from PerkinElmer Pure. Deionized water (DI) was collected from an in-house Milli-Q EQ 7000 ultrapure water purification system.

### 2.2 Experimental procedure

A total of seven feeds were prepared for temperature-swing solvent extraction with decanoic acid and diisopropylamine each used as solvents. Those include NaCl-only feeds with salt concentrations of 3.5 w/w%, 1.0 M, and 4.0 M; a selenium-only feed consisting of 500 parts per million (ppm) of SeO_4_^2−^ and 500 ppm of SeO_3_^2−^; a synthetic selenium-containing brine consisting of 500 ppm of SeO_4_^2-^ and 20 g L^−1^ Cl^−^ from NaCl; and two synthetic flue gas desulfurization wastewater samples with different concentrations of mercury composed of 20 g L^−1^ Cl^−^ from NaCl, 50 ppm of SeO_4_^2−^, 300 ppm of gypsum (CaSO_4_.2H_2_O), and 1,000 ppb or 15 ppm of Hg^2+^ from HgCl_2_.

**FIGURE 2:**
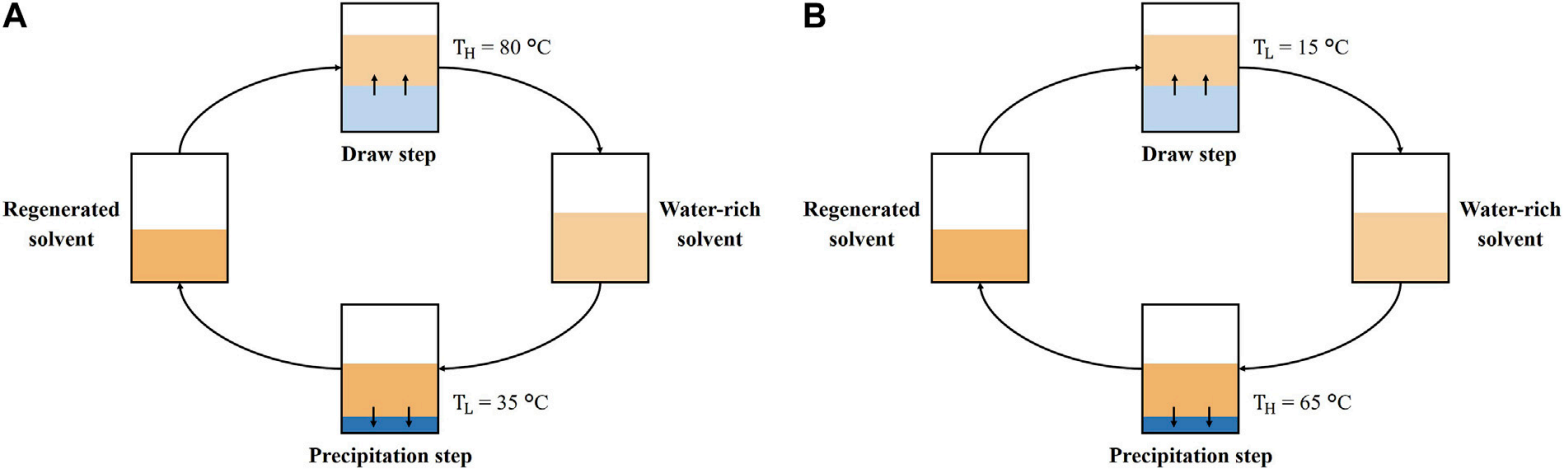
**(A)** TSSE diagram with temperatures labeled for DA extraction and **(B)** TSSE diagram with temperatures labeled for DPA extraction.

Figure 2A illustrates the procedure for TSSE with DA. In essence, 10 g of DA was transferred into a beaker and heated in an oven at 60°C until completely melted. A measure of 10 mL of the feed solution at an ambient temperature (∼24°C) and the melted DA were added to a glass vial and shaken vigorously. The vial was placed in an oil bath at a high temperature (*T*_*H*_) of 80°C for 24 h. The water-rich organic phase was pipetted into a test tube and placed in a second oil bath at a low temperature (*T*_*L*_) of 35°C for 72 h to ensure that the aqueous and organic phases split from each other. After precipitation, DA was pipetted into the original glass vial, and the aqueous phase was recovered as product water.

**FIGURE 3:**
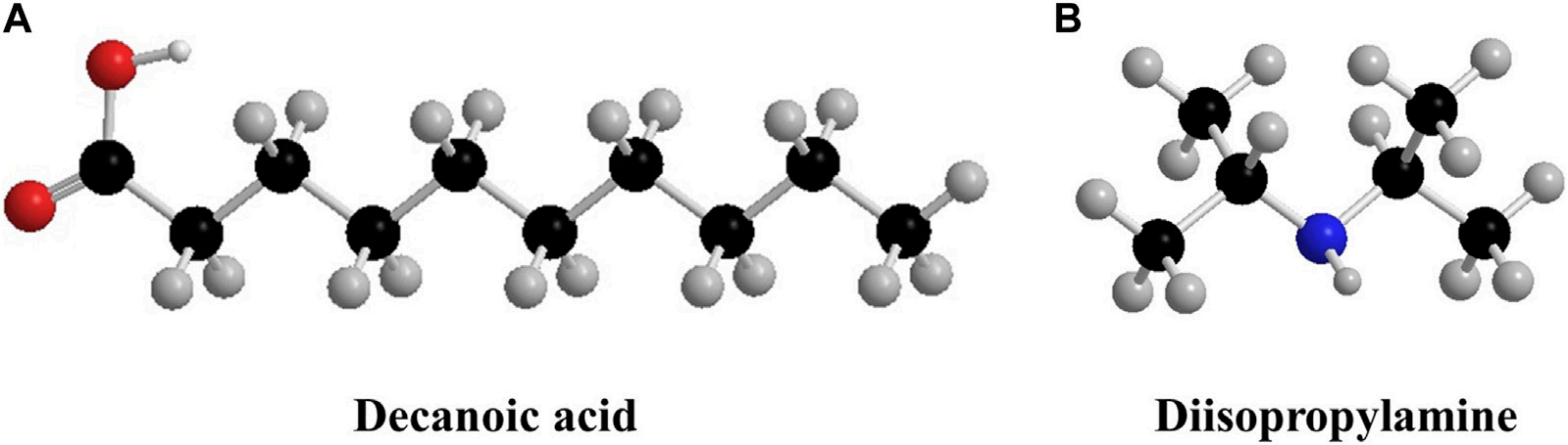
Chemical structures of **(A)** DA and **(B)** DPA.

Figure 2B describes the procedure for TSSE with DPA. A measure of 10 mL of DPA and feed were added to a glass vial at an ambient temperature (∼24°C) and shaken vigorously. The vial was placed in an oil bath at a *T*_*L*_ of 15°C for 2 h. The water-rich organic phase was pipetted into another vial and placed into an oil bath at a *T*_*H*_ of 65°C for 2 h. After precipitation, DPA was returned to the original vial, and the aqueous phase was recovered as product water. The chemical structures of DA and DPA are depicted in Figure 3.

We determined the salinity (i.e., NaCl concentration) of the product water with conductivity measurements using a Thermo Scientific Orion Star A212 conductivity benchtop meter. The calibration curves were prepared accordingly. The amount of calcium, sulfur, selenium, and mercury in the samples before and after extraction was quantified using an inductively coupled plasma-optical emission spectrometer (Varian Vista-PRO Simultaneous Axial ICP-OES) to determine their residual concentrations in the aqueous phase. The system was calibrated with a multi-element ICP calibration standard solution. Before ICP-OES measurements, all samples were diluted in 2 w/w% nitric acid added with 5 ppm of scandium as an internal standard. When essential, a PerkinElmer NexION 2000 inductively coupled plasma mass spectrometer with a collision-cell analysis capability at the University of Oklahoma Mass Spectroscopy, Proteomics & Metabolomics (MSPM) Core was used to detect trace ionic concentrations to achieve a higher measurement resolution. Specifically, given that Ar_2_ dimers interfere with Se ion signals, ICP-MS was run in the kinetic energy discrimination (KED) mode by flowing He gas through the instrument collision/reaction cell (Yamada, 2015). Before ICP-MS measurements, all samples were diluted in 2 w/w% HNO_3_ solution to a concentration of approximately ≤ 200 ppm Se/Hg. The dilutions contained 25 ppb of yttrium (Y^89^) and 50 ppb of gold (Au^197^). Y^89^ was used as a background signal ion. Au^197^ was used to stabilize ionic Hg in the solution during measurements (Allibone et al., 1999). Commercial selenium and mercury standard solutions were serially diluted and quantified to prepare calibration curves, allowing for the estimation of ionic concentration following the measurement of ion signal intensity.

## 3 Results and discussion

### 3.1 Rejection potentials of DA- and DPA-based temperature-swing solvent extraction for feed streams including NaCl, selenate/selenite, and selenate with an NaCl background

**FIGURE 4:**
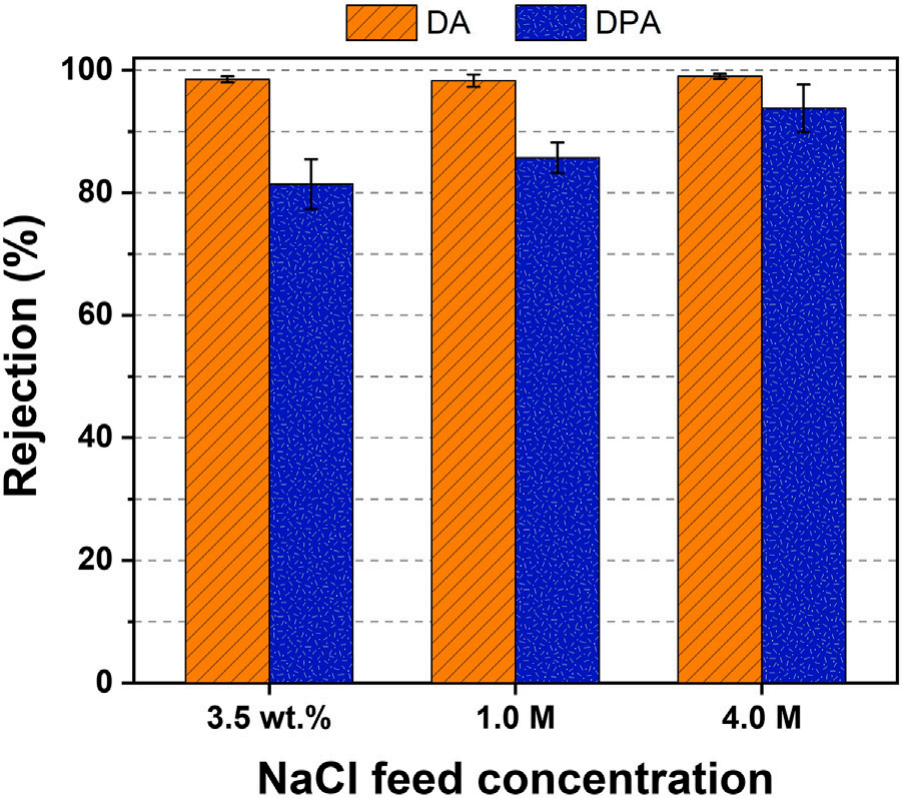
NaCl rejection of DA and DPA solvents using TSSE at different feed concentrations.

Figure 4 demonstrates the salt rejection for different NaCl feed concentrations after extraction with DA and DPA. While DPA removed a greater proportion of NaCl with more concentrated feeds ( $R_{NaCl}$ = 81.4%–93.8%), also reported in the literature (Rish et al., 2014; Boo et al., 2019), DA displayed remarkable NaCl rejection potentials, regardless of the feed concentrations (>98%). The difference in salt extraction capacities of these two solvents could probably be explained with their dielectric constants. The higher the dielectric constants of the solvent, the greater the solvent polarity and the stronger the interactions between dissociated ions and liquid. Therefore, NaCl was more preferably rejected by DA ( $\varepsilon_{DA}$ = 2.37) than by DPA ( $\varepsilon_{DPA}$ = 3.04). Note, that while the precipitation process of DA occurs at 35°C, that of DPA occurs at 65°C. Precipitating the product water from the water-rich organic phase at an elevated temperature (i.e., 65°C) may also impact the purity of the product water to a certain extent. Future studies are required to further elucidate this thermal influence on the rejection capability of TSSE solvents.

**FIGURE 5:**
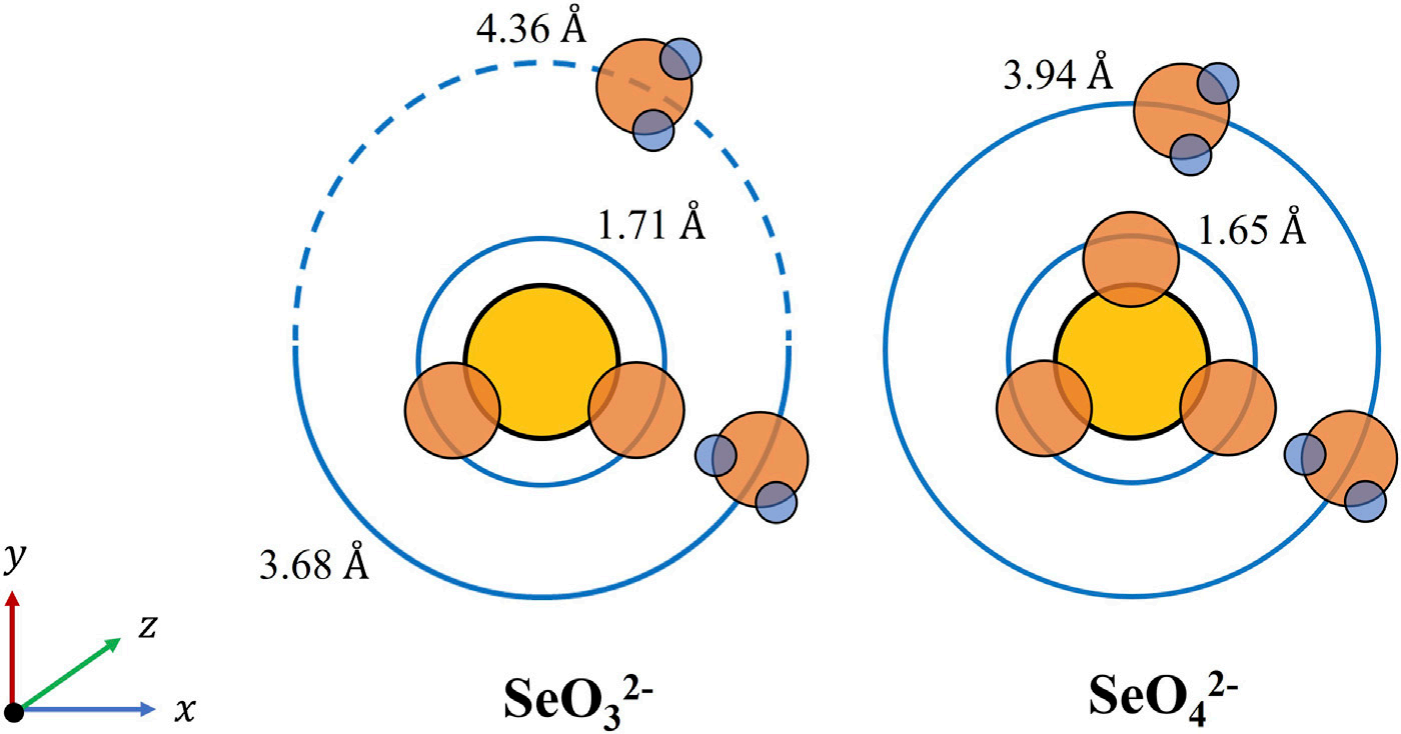
2D visualization of molecular structures and hydrated radii of a selenate and selenite anion in an aqueous solution adapted from Eklund and Persson (2014).

We then assess the rejection potential of DA and DPA against selenium oxyanions with TSSE. The molecular geometry is the principal structural difference between a selenate and selenite oxyanion. A selenate ion is comprised of four oxygens and is therefore tetrahedral, whereas a selenite ion with three oxygens is trigonal pyramidal. These structural dissimilarities bring about the distinction in the hydrated radii of these ions in the solution (Eklund and Persson, 2014). In the presence of water, hydrogen bonds form between the positive dipole of the hydrogen molecule and the negative charge of the oxygen atoms. However, selenite has a hemisphere in which there is no oxygen atom, and this causes a looser association between a selenite anion and a water molecule, creating a larger hydrated radius as a result. Indeed, as can be seen in Figure 5 (with the illustration in a two-dimensional coordinate plane), the hydrated radius of selenite in an aqueous solution is 4.36 Å vs*.* that of a selenate ion is 3.94 Å (Eklund and Persson, 2014). The larger hydrated radius of selenite may contribute to its more favorable rejection by TSSE. DA with bulky organic sections, as presented in Figure 3A, could render steric interactions and impose a higher energy barrier for selenite partition into the organic phase rather than selenate. Concurring with our theoretical hypotheses, the SeO_4_^2−^ and SeO_3_^2−^ rejection efficiencies of DA were experimentally examined to be 98.1 ± 2.9% and 98.8 ± 0.9%, respectively. Interestingly, virtually no selenium oxyanion rejection was observed for DPA in this dilute concentration range. Specifically, DPA rejected only approximately 4.14% and 8.18% of selenate and selenite, respectively, under similar testing conditions. We hypothesize that this is likely due to the hydrogen bonding interactions between the amine groups in DPA and selenium oxyanions. Unlike the carboxylate group in DA, which is under steric hindrance from the long alkyl chain, the amine group in DPA is more accessible through water and oxyanion molecules for H-bonding interactions. As selenate appeared to be more challenging to be removed using TSSE, in the subsequent steps, we assess the potential of TSSE with the chosen solvent systems in separating selenate from synthetic water samples mimicking the complex FGD waste streams.

**FIGURE 6:**
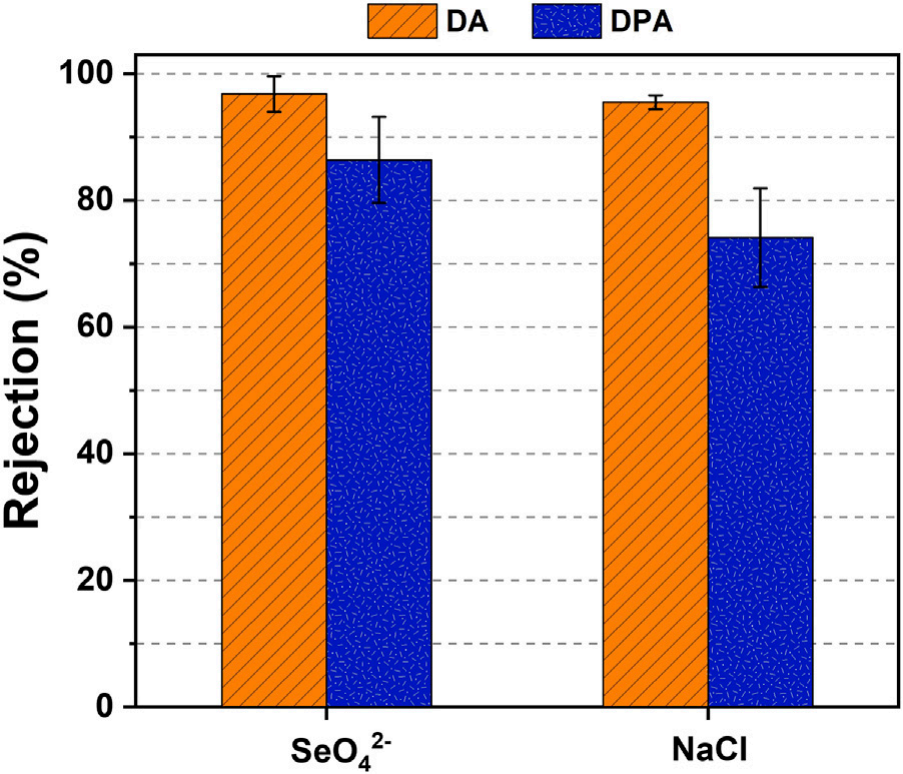
Average rejection rate of 500 ppm of selenate and 20 g L^-1^ Cl^−^ feed of DA and DPA solvents.

Figure 6 displays the ion rejection of DA and DPA from a feed stream having 500 ppm of SeO_4_^2−^ and 20 g L^-1^ of Cl^−^. DA performed a 96.8% ± 2.8% and 95.5% ± 1.1% selenate and NaCl rejection, whereas DPA showed rejections of 86.4% ± 6.8% and 74.1% ± 7.8% for selenate and NaCl, respectively. A higher rejection of selenate as compared to NaCl was observed for both solvents mainly due to the impact of hydrated ion radii on rejection (Marcus, 1988). Notably, unlike previously observed for the virtually no selenium oxyanion rejection behavior of DPA in the selenium-only feed of 500 ppm SeO_4_^2−^ and 500 ppm SeO_3_^2−^, DPA evinced the selectivity against 500 ppm of selenate in this case, i.e., when there is a coexistence of 20 g L^−1^ of NaCl. This observation marked that the ion partition between aqueous and organic phases appears to be predominately determined by the solution ionic strength (caused by 500 ppm of selenate and 20 g L^−1^ of NaCl) in preference to the concentration of the individual species in the multi-ion systems. We explain the impact of the solution ionic strength on the ion partition behaviors in aqueous and organic phases via thermodynamic models. Essentially, two phases in equilibrium follow the general equilibrium criterion given as follows (Sandler, 2017):

$${\underline{f}}_{i}^{I}\left(T,P,x^{I}\right)={\underline{f}}_{i}^{II}\left(T,P,x^{II}\right),$$ (1)

where $f_{i}^{I}$ represents the fugacity of species i in phase I (water), $f_{i}^{II}$ represents the fugacity of species i in phase II (organic solvent), T represents the temperature, P represents the pressure, and x represents the mole fraction of species i in phase I or II. Substituting the activity coefficient definition of fugacity into Eq. 1 gives

$$x_{i}^{I}\gamma_{i}^{I}\left(T,P,x^{I}\right)f_{i}\left(T,P\right)=x_{i}^{II}\gamma_{i}^{II}\left(T,P,x^{II}\right)f_{i}\left(T,P\right),$$ (2)

where $\gamma_{i}$ represents the activity coefficient of species i in phase I or II and $f_{i}$ represents the pure component liquid fugacity. The pure component liquid fugacity for a species is equivalent on both sides of Eq. 2, which is reduced to Eq. 3:

$$x_{i}^{I}\gamma_{i}^{I}\left(T,P,x^{I}\right)=x_{i}^{II}\gamma_{i}^{II}\left(T,P,x^{II}\right).$$ (3)

Equations 4, 5 introduce two terms: the mean ionic activity coefficient and the solution ionic strength. Briefly, these terms approximate the behavior of all ions within a solution rather than considering individual species.

$$\gamma_{\pm }^{v}={\left(\gamma_{i}^{*}\right)}^{v+}{\left(\gamma_{j}^{*}\right)}^{v-},$$ (4)

$$I=\frac{1}{2}\sum_{i=ions}z_{i}^{2}M_{i},$$ (5)

where $\gamma_{\pm }^{v}$ represents the mean ionic activity coefficient, ${\left(\gamma_{i}^{*}\right)}^{v+}$ represents the activity of the cations, ${\left(\gamma_{j}^{*}\right)}^{v-}$ represents the activity of the anions, I represents the ionic strength of the solution, $z_{i}$ represents the charge of ion i , and $M_{i}$ represents the concentration of ion i . With these terms, Eq. 6 introduces the Debye–Hückel limiting law, which relates the mean ionic activity to the ionic strength.

$$\ln\left(\gamma_{\pm }\right)=-\alpha \left|z_{+}z_{-}\right|\sqrt{I},$$ (6)

where α represents a parameter that depends on the solvent and temperature. Solving Eq. 6 for the mean ionic activity and applying it to Eq. 3 gives Eq. 7:

$$x_{i}^{I}*\exp\left(-\alpha \left|z_{+}z_{-}\right|\sqrt{I}\right)=x_{i}^{II}\gamma_{i}^{II}\left(T,P,x^{II}\right).$$ (7)

Equation 7 indicates that the higher the ionic strength, the greater the magnitude of the exponential expression, which in turn leads to a reduction in the mean ionic activity of the feed on the left side of Eq. 7. Consequently, a lesser ion concentration will partition into the organic phase, leading to superior solute rejections, which is consistent with the experimental results for DPA. Note, however, that the Debye–Hückel theory is only valid for dilute solutions (<0.01 M), where electrolytes completely dissociate into ions. For DPA, with a selenate rejection of roughly 4%, as discussed previously, the dynamic concentration of ions in the raffinate during the TSSE process remains low, and thus, Eq. 7 remains valid. For DA, however, we do not observe the same trend, i.e., an increase in ion rejection with ionic strength, probably because of the high ionic rejection of DA, and thus the high ionic concentration in the raffinate renders the Debye–Hückel theory invalid. Rather, the rejection behavior of DA is strongly governed by other thermodynamic barriers, such as the hindrance effect of DA, a medium-chain fatty acid that exhibits low or even negligible water miscibility (i.e., a hydrophobic deep eutectic solvent (Aparicio et al., 2023)). Further investigations into the thermodynamic behaviors of ions in biphasic systems, as a function of the concentration, are imperative.

### 3.2 Rejection potentials of DA- and DPA-based temperature-swing solvent extraction for synthetic flue gas desulfurization feed streams

**FIGURE 7:**
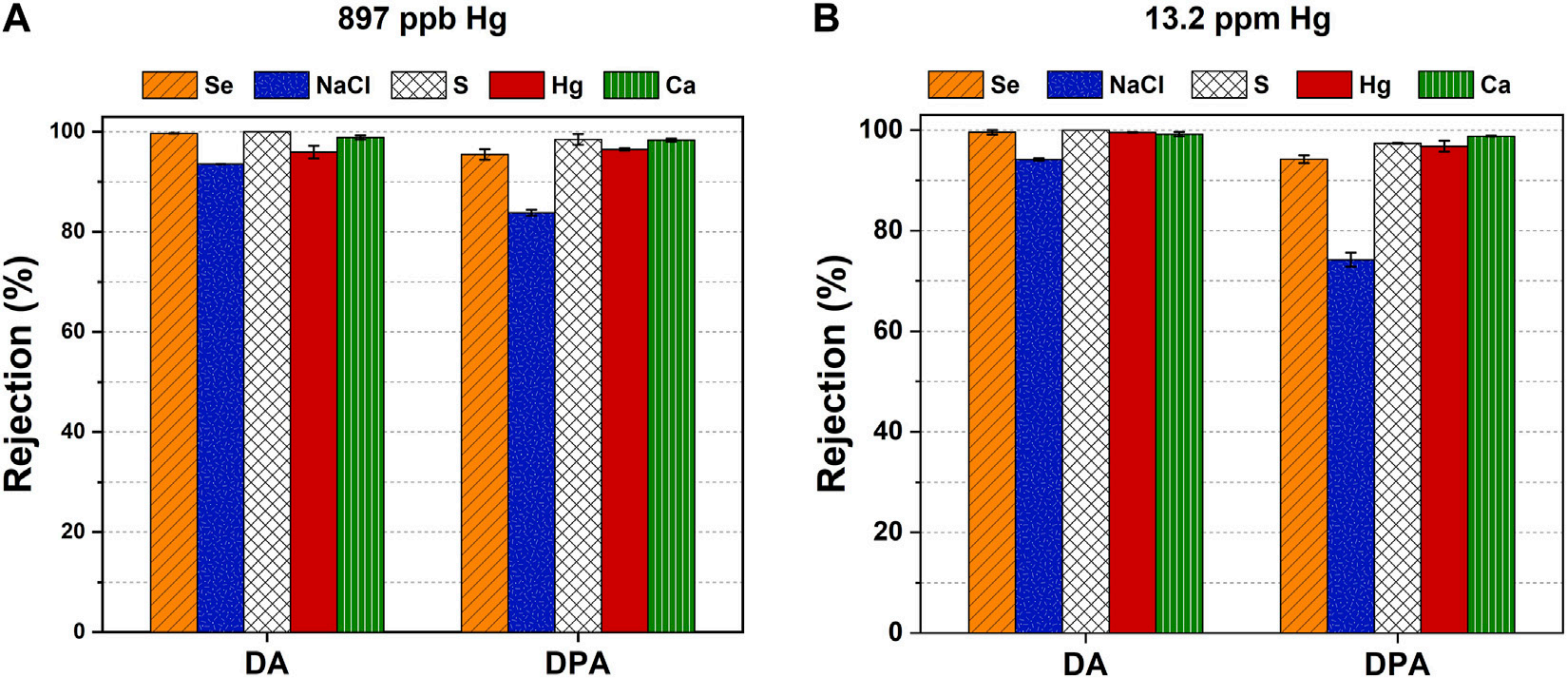
Ion rejection of TSSE with DA and DPA solvents for the two synthetic FGD streams having 300 ppm CaSO_4_, 20 g L^-1^ NaCl, 50 ppm SeO_4_^2-^, and **(A)** 897 ppb of Hg^2+^ or **(B)** 13.2 ppm of Hg^2+^.

As described previously, we assess the ion removal capability of TSSE for synthetic FGD wastewater streams having selenate that coexists with sulfate, chloride, and mercury ions. In essence, we prepared solutions having 300 ppm of gypsum, 20,000 mg L^-1^ of Cl^−^, 50 ppm of SeO_4_^2-^, and mercury with the actual concentration of 897 ppb for the first sample (FGD 1) or 13.2 ppm for the second sample (FGD 2). The results show that the medium-chain fatty acid DA performs better rejection for all ions in both FGD streams (Figure 7). Notably, the behaviors of DA and DPA toward NaCl rejection from a multiple-ion mixture remain the same as those shown in single-ion and dual-ion tests. Note that calcium, chloride, and sulfate ions may weakly interact with DA, presumably at their negatively charged carboxylate head groups and through electrostatic forces, covalent bonding, and hydrogen bonding (Yazdanian et al., 1990; Yuan et al., 2016; Gao et al., 2023; Zhang et al., 2023). Meanwhile, apart from electrostatic attraction, DPA possibly forms weak coordination complexes with inorganic ligands (i.e., chloride or sulfate) and metal centers (i.e., calcium or mercury ions) (Navarro et al., 1996; Daniele et al., 2008; Akhlaghi et al., 2015). These interactions may interfere with the ion rejection capabilities of DA and DPA to a given extent. Notwithstanding, TSSE was still able to remove >99.5% of selenium oxyanions and 96%–99.6% of mercury from a complex environment with a sixfold increase in the concentrations of calcium and sulfate vs*.* selenate and a background salinity of 20,000 mg L^-1^ NaCl. In contrast, other technologies proposed for the treatment of selenium oxyanions from FGD, such as electrochemical processes or adsorption, have still been hindered due to the lack of capabilities to effectively function without being interrupted by the background chloride level. In addition, the fact that TSSE exhibits a capability to remove traces of mercury (at 897 ppb and 13.2 ppm) reinforces the potential of this technology to provide a treatment solution for FGD wastewater toward meeting the EPA-regulated discharge levels for these toxic compounds. Seemingly, our results lay a foundational understanding essential for TSSE to effectively be integrated for metal removal and recovery and other applications with green extractants (e.g., natural deep eutectic solvents and bio-derived solvents), eventually being transformative for green and clean chemistry.

## 4 Concluding remarks

In this work, we study the potential of temperature-swing solvent extraction with decanoic acid and diisopropylamine solvents for the removal of species of concern (e.g., selenium oxyanions and mercury) from a synthetic flue gas desulfurization wastewater stream. The results show that compared to diisopropylamine, decanoic acid is more effective in rejecting ions for all cases, ranging from a simple binary system to more complex multicomponent systems with highly varied ionic concentrations, likely due to its lower dielectric constant. Furthermore, the H-bonding interaction with water and the hydrated radii of the oxyanions (i.e., selenate vs*.* selenite) along with the hindrance effects caused by the molecular bulkiness and hydrophobicity (or lipophilicity) of the solvents play important roles in the favorable rejection of TSSE. It implies that one can tune the selectivity of TSSE with appropriately selected solvents having specific chemical descriptors (e.g., functional groups or ligands). Of note, while DA rejects selenium oxyanions significantly, DPA appears to be more sensitive to the total ionic strength of the solution. Specifically, while DPA can only reject 4%–8% of selenate and selenite from 500 ppm mixed-Se solutions, its rejection rate toward selenate was increased to approximately 86% when there was a coexistence of 20 g L^-1^ of NaCl. This result is corroborated with our thermodynamic analyses, implying that a higher ionic strength can lead to a reduction in the mean ionic activity in the feed, thereby reducing the amount of ions partitioning into the organic phase, culminating in a superior solute rejection. Furthermore, from this test, although the concentration of NaCl in the feed stream is 400-fold higher than that of selenate, the two solvents exhibit a higher rejection rate of selenate than that of NaCl. Lastly, TSSE shows a great ion-separating performance from synthetic FGD wastewater streams. Specifically, TSSE can remove >99.5% selenium oxyanions and 96%–99.6% mercury from the discharge stream with the coexistence of sulfate at a six-fold increase in the concentration. In summary, we demonstrated that TSSE is promising either as a standalone or a pre-treatment technology to alleviate Se and Hg from FGD discharge streams, helping CFPP facilities to comply with EPA regulations. TSSE is especially efficient and cost-effective in processes where the coexistence of highly varied concentrations of chloride and sulfate is of concern for other technologies due to their low tolerance of these species.

## Data Availability

The original contributions presented in the study are included in the article/Supplementary Material; further inquiries can be directed to the corresponding author.
